# Zetapalatopharyngoplasty in treatment of obstructive sleep apnea: a 10-year retrospective study

**DOI:** 10.1016/j.bjorl.2026.101796

**Published:** 2026-03-19

**Authors:** Alan Rodrigues de Almeida Paiva, Mauro Becker Santos Vieira, Roberto Eustáquio Santos Guimaraes, Ana Paula Alves Pereira, Yuri Alexandre Mota Amaral, Maria Clara Argolo Costa, Nayane Oliveira Pio

**Affiliations:** a Hospital Felicio Rocho, Department of Othorinolaryngology, Belo Horizonte, MG, Brazil; b Universidade Federal de Minas Gerais, Department of Surgery, Belo Horizonte, MG, Brazil; c Hospital Socor, Department of Otorhinolaryngology, Belo Horizonte, MG, Brazil

**Keywords:** Sleep apnea, Obstructive, Pharyngeal muscles, Polysomnography, Surgery, Quality of life

## Abstract

**Objective:**

To evaluate the efficiency of the Zetapalatopharyngoplasty (ZPFP) technique for Obstructive Sleep Apnea (OSA).

**Methods:**

This retrospective study included 36 adult patients treated with ZPFP between 2012 and 2023. Clinical, polysomnographic data, and subjective assessments using the Epworth Sleepiness Scale (ESS) and the Sleep Apnea Quality of Life Index (SAQLI) were analyzed. Surgical success was defined as a ≥50% reduction in the Apnea-Hypopnea Index (AHI) or a postoperative AHI <20. The mean follow-up duration was approximately 40-months.

**Results:**

The mean AHI decreased from 34.22 to 19.28 events/hour (p = 0.009), while desaturation time dropped from 51.09 to 17.71 min (p < 0.001). ESS scores improved from 12.36 to 6.25 (p = 0.002), indicating reduced daytime sleepiness. According to the SAQLI, 97% of patients experienced moderate to significant improvements in quality of life. Early complications included tonsillar bleeding (2.8%) and dysphagia (5.6%), while late complications involved pharyngeal reflux (8.3%) and voice changes (8.3%). Nosevere adverse events occurred.

**Conclusion:**

ZPFP demonstrated favorable clinical and polysomnographic outcomes, offering a safe and effective surgical alternative for CPAP-intolerant OSA patients. Future prospective studies should assess its long-term effectiveness in reducing cardiovascular risks associated with untreated OSA.

**Level of evidence:**

3.

## Highlights

• ZPFP surgery significantly reduced AHI and T90 scores, improving OSA severity.
• A 10-year follow-up period evaluating palatoplasty outcomes.
• Patients demonstrated significant enhancements in SAQLI and ESS scores.
• Postoperative ZPFP complications were mild, with no mortality or severe adverse events.
• Patients’ non-adherent to CPAP benefited significantly from ZPFP surgical intervention.

## Introduction

Obstructive Sleep Apnea (OSA) is a chronic condition that compromises quality of life, causes cognitive dysfunction, and increases mortality risk.^1^ In OSA, intermittent collapses of the upper airways during sleep result in hypercapnia and chronic hypoxia.^2^ Over the long term, this nocturnal hypoxic burden is associated with systemic consequences, including arterial hypertension, metabolic dysregulation, neurocognitive decline, and increased risk of cardiovascular disease.^1^^,^^3^

The treatment for OSA depends on the severity of the disease. Lifestyle changes, weight loss, and mandibular advancement devices are recommended for mild cases.^4^ In patients with moderate to severe OSA, Continuous Positive Airway Pressure (CPAP) therapy is the first-line treatment, as regular use (≥4-hs per night) results in a significant reduction in cardiovascular events.^5^ However, clinical trials indicate that the population-level benefits are modest due to poor adherence to prolonged CPAP use, with non-adherence rates ranging from 35% to 64%.^6^^,^^7^

Although noninvasive therapies are effective, low adherence leads to many patients receiving inadequate treatment, which in turn leads to disease progression. Studies show that surgical interventions for OSA in CPAP non-adherent patients are effective and cost-effective, providing substantial benefits such as reduced cardiovascular risk and lower long-term healthcare costs.^8^^,^^9^

OSA surgeries are performed based on the level of obstruction in the upper airways, ranging from nasal interventions to the hypopharynx, with the choice of procedure depending on the patient’s anatomy.^10^ Among the obstruction sites, the velopharynx is the most frequently affected.^11^ Early surgical approaches focused on palatoplasty, with Uvulopalatopharyngoplasty (UPPP) widely used over the past decade. Randomized studies report 30%–80% success rates in reducing the Apnea-Hypopnea Index (AHI).^12^^,^^13^

Despite its impact, UPPP is not widely accepted due to high late complication rates, including chronic dysphagia in 29%–36% of cases and severe issues like velopharyngeal insufficiency and nasopharyngeal stenosis.^14^^,^^15^ Technical variations have emerged to minimize risks, including Zetapalatopharyngoplasty (ZPFP), introduced by Vieira et al. in 2001.^16^ ZPFP offers advantages, such as fewer complications and good clinical outcomes, though available data remain limited for validation.

In this context, this study aims to evaluate the efficacy of ZPFP using objective polysomnographic data and subjective quality-of-life questionnaires in patients who have undergone surgery in the last 10-years.

## Methods

### Study design

This retrospective study included adult patients treated for OSA using the ZPFP technique at Hospital Felicio Rocho in Belo Horizonte, Minas Gerais, Brazil. Data were collected by reviewing medical records, patient-provided documents, and in-person questionnaires. The study was registered with and approved by the Research Ethics Committee at Hospital Felicio Rocho under approval number 5.738.809.

### Sample size and selection

All patients who underwent ZPFP between 2012 and 2023 were initially considered eligible for the study. Patients were selected based on awake endoscopic findings indicating obstruction confined to the velopharyngeal region, as evidenced by the Müller maneuver or the presence of excess palatal soft tissue. Patients who underwent simultaneous nasal or sinus procedures for coexisting disorders during the same operative session were included in the study. The final selection included only patients diagnosed with OSA, according to the current criteria of the American Academy of Sleep Medicine (AASM), and who were refractory to non-invasive treatments.^9^

Inclusion criteria comprised previous ZPFP surgery, absence of any prior or subsequent surgical intervention for OSA, signed informed consent, and availability of medical records or original documents containing polysomnographic results obtained both preoperatively and at least six months postoperatively. Exclusion criteria included incomplete medical records, absence of a confirmed OSA diagnosis, lack of polysomnographic data, and patients who did not provide informed consent.

### Surgical technique

**Fig. 1:**
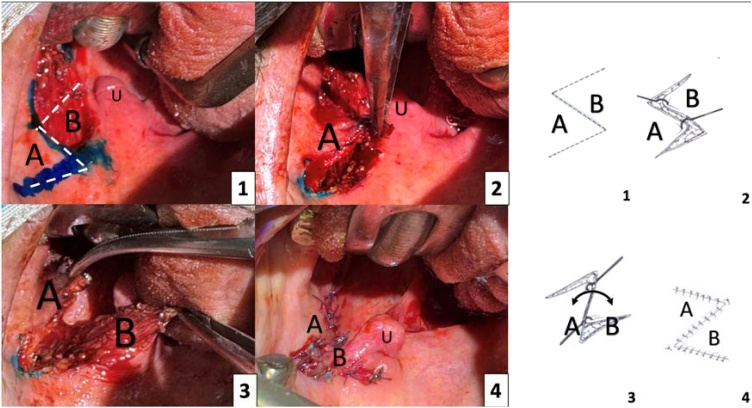
(A) Anterior flap; (B) Posterior flap; (U) Uvula. Photo 1 illustrates the planned incisions for the anterior flap along the palatoglossal arch (highlighted with methylene blue) and for the posterior flap along the palatopharyngeal arch. Photo 2 shows the dissection of the anterior flap, labeled as A. Photo 3 presents both flaps fully dissected and held with forceps. Photo 4 demonstrates the transposition of the flaps and their fixation through suturing.

The procedure is performed under general anesthesia, with the patient's head in extension and oral cavity exposure achieved using a mouth gag. It begins with a bilateral tonsillectomy. The surgical technique involves the creation of two mucosal-muscular flaps to execute a Zetapalatopharyngoplasty, characterized by the transposition of tissue flaps in a “Z” configuration. The anterior flap is created along the palatoglossal arch, with an incision limited to the mucosa of the soft palate, extending laterally and superiorly from the upper pole of the tonsillar fossa toward the pterygoid hamulus, followed by careful dissection, as illustrated in Fig. 1. A second incision is made at the junction between the middle and lower thirds of the posterior tonsillar pillar, elevating this flap together with the underlying fibers of the palatopharyngeal muscle. Once both flaps are mobilized, the ZPFP is completed by transposing and suturing the inferior flap to the upper soft palate while the anterior flap is repositioned and anchored inferiorly (Fig. 1).^16^

### Definition of variables

#### Polysomnography

The polysomnography used in the study was type 1, performed both pre- and post-operatively. Type 1 polysomnography involves the monitoring of electroencephalography, electrooculography, electromyography, oxygen saturation, electrocardiography, airflow (nasal and oral), respiratory effort (thoracic and abdominal), snoring, and body position. The snoring score exhibited variability across sleep laboratories and was therefore excluded from the analysis. The AHI was standardized according to AASM guidelines.^17^ Apnea was defined as a reduction in airflow to less than 10% of the mean flow for more than 10 seconds, associated with a 4% oxygen desaturation or complete cessation of airflow. Hypopnea was defined as a more than 30% reduction in airflow for over 10 seconds with a 4% oxygen desaturation. OSA severity was classified based on the AHI: mild (5–14), moderate (15–30), and severe (>30).^17^^,^^18^ Surgical success was defined as a reduction in AHI of at least 50% or a post-operative AHI of less than 20.^13^ Desaturation was recorded as the minutes spent with oxygen saturation below 90% (T90) during polysomnography.

#### Daytime sleepiness

Quality of life and daytime sleepiness questionnaires were applied in person at the respective hospitals. Daytime sleepiness was assessed using the Epworth Sleepiness Scale (ESS), which consists of eight situations where patients rate their likelihood of falling asleep during the day, from 0 (no chance) to 3 (high chance). The total score is the sum of the ratings for each item. Patients completed the questionnaire both before surgery and at the time of the study. According to Johns MW, the ESS indicates that scores from 1 to 6 are considered normal, 7–9 indicate moderate sleepiness, and scores of 10 or higher indicate excessive daytime sleepiness.^19^

### Quality of life assessment

The Sleep Apnea Quality of Life Index (SAQLI) evaluates OSA patients' quality of life after treatments like CPAP, surgery, oral appliances, or weight loss. It includes 35 items in four domains: daily functioning, social interactions, emotional functioning, and symptoms, scored on a 7-point Likert scale, with higher scores indicating better quality of life. A fifth domain covers treatment-related symptoms and subjective improvement perception. The questionnaire was translated and validated for Portuguese.^20^

The minimal clinically important difference (MCID) for SAQLI interprets quality-of-life changes post-treatment, considering surgical effects and overall well-being. Domain changes were calculated by averaging item scores, with a total score adjusting for treatment-related symptoms. Changes were classified as “No change” (−1 to 1), “Minimal important difference” (1.1–3 improvement or −1.1 to −3 worsening), “Moderate difference” (3.1–5 improvement or −3.1 to −5 worsening), and “Large difference” (5.1–7 improvement or −5.1 to −7 worsening).^21^

### Surgical complications

All complications occurring in the study patients were reviewed through medical records and patient interviews. Early complications were defined as those occurring in the perioperative period up to 30-days post-surgery. Late complications were defined as those occurring after the 31st day post-procedure.

### Statistical analysis

The statistical analysis began with a descriptive evaluation of the variables, considering the distribution of continuous and categorical variables. The mean and standard deviation were used for continuous variables with normal distribution, while the median and interquartile range (P25%‒P75%) were applied for non-normal distributions. Paired Student's *t*-test was used to compare variables between pre- and post-operative periods, and an unpaired Student's *t*-test was used for comparison between independent groups. Pearson’s chi-square test was applied for categorical variables. Multivariate analysis was conducted using binary logistic regression, with a 95% Confidence Interval and a significance level of p < 0.05. Model fit was assessed using the Hosmer & Lemeshow test.

## Results

Initially, 119 patients who underwent ZPFP were selected. Of these, 48 were excluded for not having a second polysomnography, 23 for lack of updated contact information or failure to respond to available communication methods, and 12 for insufficient data in their medical records or original documents. Thus, the sample was composed of 36 individuals.

The sample had a mean age of 49.78 ± 10.78 years, with a predominance of adults (75%) and males (69.4%). The mean BMI was 28.01 ± 3.38 kg/m^2^, with 30.6% classified as obese. Approximately 36.1% had hypertension, and most had at least one comorbidity (Table 1).

**Table 1:** Demographic and clinical characteristics of the sample.

Variable	Category	n (%)
Age (years)	‒	49.78 ± 10.78^a^
Age Range	Young Adult (19–35)	03 (8.3)
	Adult (36–59)	33 (91.0)
Gender	Female	11 (30.6)
	Male	25 (69.4)
BMI	‒	28.01 ± 3.38^a^
Obesity	Yes	11 (30.6)
	No	25 (69.4)
Hypertension	Yes	13 (36.1)
	No	23 (63.9)
Comorbidities	None	13 (36.1)
	1	15 (41.7)
	2	08 (22.2)
Mallampati scale	1 + 2	18 (50.0)
	3 + 4	18 (50.0)
Tonsils size	1 + 2	33 (91.7)
	3	03 (8.3)
Septum & Turbinectomy	Yes	23 (63.9)
	No	13 (36.1)
Sinus Surgery	Yes	08 (22.2)
	No	28 (77.8)
Friedman scale	1	02 (5.6)
	2	17 (47.2)
	3	17 (47.2)
Postoperative Time (years)	‒	5.25 ± 3.48^a^
Postoperative PSG Time (months)	‒	40.11 ± 35.84^a^
Postoperative Weight	Decreased	13 (39.4)
	Maintained/Increased	20 (60.6)
Surgical Success	Yes	21 (58.3)
	No	15 (41.7)
Surgeon	>20-years	26 (72.2)
	<10-years	10 (27.8)

PSG, Polysomnography; BMI, Body Mass Index.

a Mean ± Standard Deviation.

Comparative analyses between pre- and post-operative periods showed significant changes in respiratory variables. The desaturation time decreased from an average of 51.09 min to 17.71 min (p < 0.0001). The proportion of patients with minimum saturation below 90% decreased from 94.1% preoperatively to 85.3% postoperatively (-9.4%, p = 0.3753), not reaching statistical significance. Conversely, the percentage of patients without severe desaturation increased from 5.9% to 14.7% (+149.2%), as shown in Table 2.

**Table 2:** Preoperative and postoperative results.

Measure	Preoperative	Postoperative	Variation (%)	p-value
Weight	81.18 ± 13.91^a^	81.12 ± 13.70^a^	−0.07%	0.9401^b^
Minimum Saturation				
Severe (< 90%)	32 (94.1)	29 (85.3)	−9.40%	0.375^d^
Not severe	02 (5.9)	05 (14.7)	+149.15%	
Desaturation Time	51.09 ± 52.37^a^	17.71 ± 27.50^a^	−65.33%	<0.001^c^
AHI	34.22 ± 22.24^a^	19.28 ± 14.91^a^	−43.63%	<0.0001^d^
AHI Classification				
Mild	08 (22.2)	16 (44.4)	+100.00%	0.0093^d^
Moderate	09 (25.0)	14 (38.9)	+55.60%	
Severe	19 (52.8)	06 (16.7)	−68.40%	
Epworth	12.36 ± 3.56^a^	6.25 ± 3.00^a^	−49.43%	0.001^d^
Epworth Classification				0.0023^d^
Normal	01 (4.2)	12 (50.0)	+1095.24	
Moderate	05 (20.8)	08 (33.3)	+60.58	
Excessive	18 (75.0)	04 (16.7)	−77.73	

a Mean ± Standard Deviation.

b Paired *t*-test.

c Wilcoxon test.

d McNemar test.

The AHI reduced from 34.22 to 19.28 (p = 0.0093), and the ESS dropped from 12.36 to 6.25 (p = 0.0023). The percentage reductions in means ranged from −43.63% to −65.33% (Table 2).

The SAQLI score had a mean of 5.61 ± 0.62. Based on the calculation of the MCID, only one patient showed a minimal difference, while all others exhibited a moderate improvement following treatment, accounting for approximately 97% of the patients. No patient experienced a deterioration in quality of life or a large difference.

**Fig. 2:**
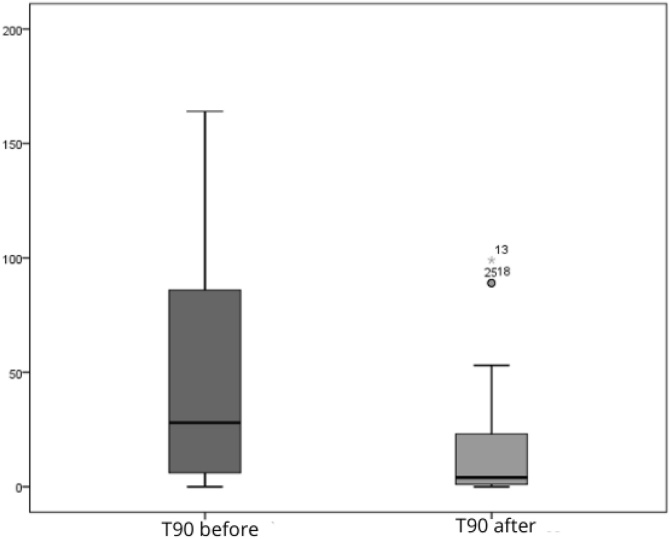
Box-plot of pre- and post-surgery saturation time.

**Fig. 3:**
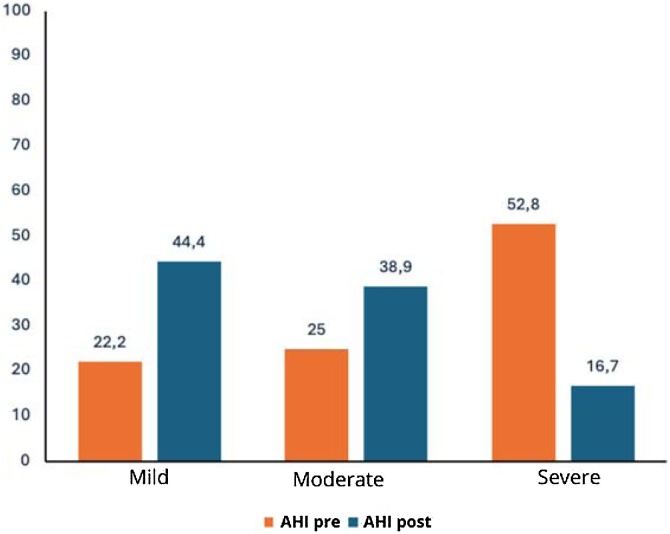
Bar Graph showing the percentages of the AHI classification before and after surgery.

**Fig. 4:**
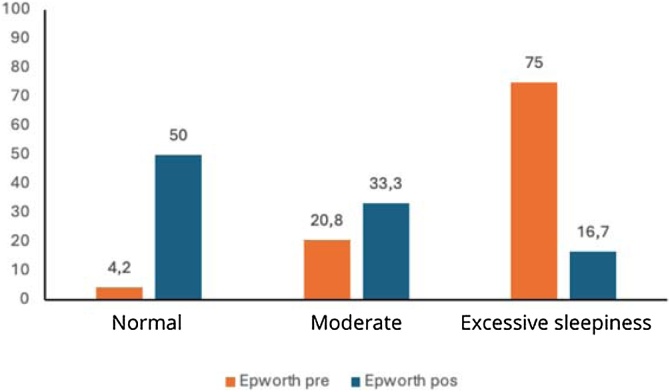
Bar Graph showing Epworth percentages before and after surgery.

The boxplot for desaturation time (Fig. 2) shows a reduction in post-operative values, indicating a concentration of shorter times. The AHI bar graph (Fig. 3) illustrates a shift in patient distribution to lower severity categories after surgery. Fig. 4 demonstrates a decrease in the ESS score, with an approximate 50% reduction in excessive daytime sleepiness after the procedure.

The multivariate logistic regression model revealed that T90 was significantly associated with surgical success (p = 0.023). Patients with desaturation time less than 12 min had 8.67 times higher odds of surgical success compared to those with desaturation time ≥12 min (OR = 8.67; 95% CI 1.35; 55.73). Pre-operative AHI, however, showed no significant association with surgical success (p = 0.804), with an Odds Ratio (OR) of 1.26 (95% CI: 0.203; 7.836), as shown in Table 3. The Hosmer & Lemeshow test indicated a good model fit (p = 0.538).

**Table 3:** Logistic regression model adjustment results for surgical success evaluation.

Variables	Surgical Success		OR	95% CI / OR	p-value
	Yes (n = 21)	No (n = 15)			
Desaturation Time (Pre)					
<12 min	03	09	8.67	1.35 / 55.73	0.023
≥12 min	18	06	1		
AHI (Pre)					
<15	03	06	1.26	0.203 / 7.836	0.804
≥15	18	09	1		

Hosmer & Lemeshow test (p = 0.538).

No statistically significant differences were observed in surgical success, postoperative AHI, T90, SAQLI, or ESS concerning surgeon, presence of supine-dependent apnea, Mallampati index, tonsil size, or Friedman classification.

Septoplasty and turbinectomy surgery significantly improved postoperative desaturation (p = 0.0081), with 95.7% of patients demonstrating improvement. The variables sinus surgery, post-operative weight, and ESS did not show statistically significant differences (p = 0.4221, p = 0.4891, and p = 0.8501, respectively). Regardless of these interventions, most patients showed improvements in desaturation and ESS (Table 4).

**Table 4:** Results of the comparison of desaturation time in the post and pre periods between the patients' surgical variables.

Variable	Desaturation Time Difference			p-value
	Worse	Maintained	Improved	
Septum & Turbinectomy				
Yes	01 (4.3)	00 (0.0)	22 (95.7)	0.008^a^
No	02 (15.3)	05 (38.4)	06 (46.1)	
Sinus Surgery				
Yes	00 (0.0)	00 (0.0)	08 (100.0)	0.422^a^
No	03 (10.7)	05 (17.8)	20 (71.4)	
Post-Surgery Weight				
Increased	02 (15.4)	02 (15.4)	09 (69.2)	
Maintained	00 (0.0)	01 (14.3)	09 (85.7)	0.489^a^
Decreased	01 (7.7)	00 (0.0)	12 (92.3)	
Epworth				
Improved	02 (12.5)	02 (12.5)	12 (75.0)	0.850^a^
Maintained	01 (16.7)	00 (0.0)	05 (83.3)	
Worse	00 (0.0)	00 (0.0)	01 (100.0)	

a Exact Pearson Chi-Square test.

Early complications were observed in some patients, including tonsillar bleeding in one patient (2.8%), persistent dysphagia (5.6%), and velopharyngeal insufficiency (16.7%). Late complications included pharyngeal reflux symptoms in three patients (8.3%), a sensation of constriction (5.6%), voice changes (8.3%), foreign body sensations (8.3%), and excess mucus (2.8%). No cases of mortality, emergency tracheostomy, or nasopharyngeal stenosis were recorded (Table 5).

**Table 5:** Surgical complications.

Complication	Type	Frequency (n)	Percentage (%)
Early Tonsillar Bleeding	Early	01	2.8
Persistent Dysphagia	Early	02	5.6
Velopharyngeal Insufficiency	Early	06	16.7
Pharyngeal Reflux Symptoms	Late	03	8.3
Constriction Sensation	Late	02	5.6
Vocal Changes	Late	03	8.3
Velopharyngeal insufficiency	Late	00	0.0
Foreign Body Sensation	Late	03	8.3
Excess Mucus	Late	01	2.8
Mortality, emergency tracheostomy or nasopharyngeal stenosis	Late	00	0.0

## Discussion

This is the first study evaluating the objective effects of ZPFP in the treatment of OSA through polysomnography and the first to include patients with more than 10-years of follow-up among existing palatoplasty procedures. The efficacy of the technique, assessed by both objective and subjective data, proved clinically relevant, with a surgical success rate of approximately 58% based on AHI and a reduction in nocturnal desaturation time of 65%. Additionally, quality of life and daytime sleepiness assessments showed significant clinical improvement.

Moderate to severe OSA affects about 22% of the global population, according to Benjafield et al.^22^ In Brazil, studies in São Paulo indicated a 32.8% prevalence.^23^ Untreated OSA increases the risk of cardiovascular events, including myocardial infarction, stroke, and exacerbation of type 2 diabetes.^24^ Sleep fragmentation reduces restorative sleep phases, causing daytime sleepiness, cognitive deficits, impaired motor coordination, and compromised executive functions.^25^ Thus, OSA poses a significant public health challenge, increasing healthcare demands and costs.^26^

Surgical interventions offer an alternative in the treatment when CPAP adherence is low. Weaver et al. found a 30% higher survival rate with UPPP compared to CPAP in older men.^27^ Martin et al. showed that sleep surgery provides greater survival and reduced cardiovascular morbidity over extended follow-ups.^28^ Abdelwahab et al. reported lower healthcare costs and utilization among patients treated surgically versus those using CPAP.^29^

Polysomnography remains the gold standard for diagnosing OSA. Historically, the AHI has been strongly associated with increased morbidity and mortality. For instance, a meta-analysis by Ge et al. found AHI to be an independent predictor of both cardiovascular and all-cause mortality, showing hazard ratios of 2.65 and 1.90, respectively, in cases of severe OSA.^24^ Furthermore, long-term research consistently confirms a heightened risk of myocardial infarction and stroke linked to elevated AHI.^30^ More recently, nocturnal desaturation has emerged as a significant predictor of adverse outcomes in OSA. Specifically, T90 has been identified as an independent predictor of cardiovascular events. A cohort study by Xu et al., reported a hazard ratio of 1.41 for cardiovascular events associated with T90.^31^ Other indices related to hypoxemia, such as minimum oxygen saturation and the oxygen desaturation index, have also been linked to an increased risk of myocardial infarction and mortality.^32^^,^^33^

In ZPFP-treated patients, the average desaturation time significantly reduced from 51.09 ± 52.37 min preoperatively to 17.71 ± 27.50 min postoperatively, representing approximately a 65% decrease. When evaluating lowest oxygen saturation, the proportion of patients without severe desaturation more than doubled, increasing from 5.9% preoperatively to 14.7% postoperatively (a 149.2% relative increase). This technique demonstrated improvement in cardiovascular risk parameters in OSA patients. Furthermore, multivariate logistic regression identified T90 below 12 min as the sole significant predictor of surgical success, suggesting its potential utility in patient selection. Nevertheless, patients with higher T90 may still derive benefits from surgery, such as enhanced CPAP tolerance or reduced pressure requirements.

A recent meta-analysis of 18 palatoplasty techniques reported a mean short-term success rate of 67.5% at 3–12 months postoperatively.^13^ However, long-term efficacy diminishes: He et al. observed that success following Uvulopalatopharyngoplasty (UPPP) declined from 67.3% to 44.35% beyond 34-months of follow-up.^34^ Likewise, another long-term series documented surgical success in 53% of patients after the first postoperative evaluation, 54% at 34 months, and 50% at 48 months.^35^ In our study, with an average polysomnographic follow-up of 40-months, mean AHI decreased by 14.94 events/hour, yielding a surgical success rate of 58.3%, comparable to other palatoplasty techniques. Such sustained reductions in AHI have been linked to an approximately 24.8% decrease in cardiovascular disease risk.^36^

Including nasal surgery alongside other sleep surgery approaches improves OSA outcomes. Yuan et al. showed that extended UPPP combined with nasal surgery improved AHI, ESS, and oxygen saturation, with an 87.10% overall response rate.^37^ A multicenter prospective study involving 735 patients from eight countries demonstrated that the addition of nasal surgery to multilevel palate and/or tongue procedures significantly improved outcomes in OSA treatment. Patients who underwent combined nasal and sleep surgeries showed greater reductions in AHI and ESS scores, with a higher surgical success rate (68.2% vs. 55.0%, p = 0.002), and larger percentage changes in AHI (33.7%) and ESS (37%) compared to those without nasal intervention (p ≤ 0.001).^38^

Excessive daytime sleepiness has been widely recognized as a cardinal symptom of OSA since the earliest reports in the 1970s.^17^^,^^19^ Several clinical trials have demonstrated that OSA treatment significantly improves both self-reported and objective measures of sleepiness.^39^ While the severity of OSA assessed objectively by AHI is associated with improvements in sleepiness in some studies, baseline sleepiness severity evaluated by the ESS is a better predictor of improvement, highlighting the importance of individual response to OSA as a marker of disease severity.^18^ Rashwan et al. compared different palatoplasty techniques and found that Barbed Reposition Pharyngoplasty (BRP) resulted in a mean ESS reduction of 5.52 ± 4.1 points, while expansion sphincter pharyngoplasty achieved a reduction of 4.84 ± 3.3 points, and UPPP showed a reduction of 1.36 ± 1.9 points.^40^ In the present study, ZPFP resulted in an ESS reduction of approximately 6 points and decreased the prevalence of excessive daytime sleepiness by 77.7% among surgically treated patients.

The SAQLI assesses sleep apnea-related quality of life. Studies by Avlonitou et al. and Batool-Anwar et al. showed significant SAQLI improvements with CPAP therapy.^41^^,^^42^ Additionally, research by Flemons and Reimer confirmed the high validity and sensitivity of SAQLI in measuring quality-of-life enhancements.^43^ In the study by Parish and Lyng, the average SAQLI score for patients before CPAP therapy was 4.9 ± 1.2, improving after treatment.^44^ This is the first palatoplasty study assessing SAQLI in post-intervention quality of life, with an average score of 5.61 ± 0.62. MCID revealed that 97% of patients showed moderate overall quality-of-life improvement, with only one patient showing minimal change.

Thus, ZPFP surgical intervention improved daytime sleepiness and patient quality of life, as evidenced by reduced ESS scores and positive SAQLI outcomes, resulting in high patient satisfaction post-surgery.

The central focus of current palatoplasty techniques involves the palatopharyngeus muscle.^13^^,^^45^ In the method used in this study, the muscle is sectioned to reduce the oropharyngeal constrictive tension it generates. However, this surgical step can lead to complications such as voice changes and dysphagia, although these occur in a minority of cases.^45^ Recently, alternative techniques that preserve the integrity of the palatopharyngeus muscle, such as suspension pharyngoplasty, have been developed, demonstrating favorable results at 12-months of follow-up.^46^ Nevertheless, even when the muscle is preserved and only repositioned rather than transected, postoperative speech alterations and dysphagia can still occur.^47^

These potential complications highlight the importance of balancing efficacy and invasiveness in palatal surgeries. In this context, the modified UPPP aimed to reduce invasiveness. Friberg et al. reported mild long-term issues such as globus sensation, mucus production, and swallowing changes in 31%–38% of cases.^48^ In BRP, dysphagia (5.6%), bleeding (1.5%), and velopharyngeal insufficiency (1.5%) occurred.[49] This study reported one case of postoperative bleeding and a 6% persistent dysphagia rate. Late complications included pharyngeal reflux, throat constriction, and voice changes in 2.7%–8% of cases, with no severe outcomes like emergency tracheostomy or nasopharyngeal stenosis. Thus, ZPFP presents complication rates comparable to minimally invasive palatoplasty techniques.

### Limitations

Although this investigation provides insights into ZPFP outcomes, certain methodological constraints warrant cautious interpretation. Its retrospective design may have introduced selection bias by excluding patients who did not return for follow-up assessments. In Brazil, the high cost and limited availability of polysomnography restricted the number of participants who could undergo objective evaluation, potentially amplifying sampling bias. The modest sample size and variable follow-up duration further limit the generalizability of our findings. Patient-reported quality-of-life outcomes are also vulnerable to recall bias, as subjective factors can influence symptom recollection. Lastly, by focusing exclusively on CPAP-intolerant individuals without stratifying by OSA severity, the study may not fully capture the clinical spectrum of the disorder. Future research should adopt prospective, controlled designs with broader patient inclusion and comprehensive long-term follow-up.

## Conclusion

The ZPFP reduced the apnea-hypopnea index by 14.94 events/hour and cut nocturnal desaturation time by 65% at a mean follow-up of 40 months. Daytime sleepiness and quality-of-life scores improved, and the overall success rate was 58.3%, with complication rates similar to other palatoplasty techniques. Postoperative T90 below 12 min emerged as an independent predictor of success. ZPFP may be a treatment option for CPAP-intolerant patients; randomized trials are needed to compare its outcomes with those of alternative surgical approaches.

## ORCID ID

Alan Rodrigues de Almeida Paiva: 0000-0001-8092-0146

Ana Paula Alves Pereira: 0009-0006-2787-9459

Yuri Alexandre Mota Amaral: 0009-0008-3058-0209

Maria Clara Argolo Costa: 0000-0003-1852-3347

## Funding

No funding was secured for this study.

## Data availability statement

The authors declare that all data are available in repository.

## Declaration of competing interest

The authors declare no conflicts of interest.
